# Perspective: Cancer Patient Management Challenges During the COVID-19 Pandemic

**DOI:** 10.3389/fonc.2020.01556

**Published:** 2020-08-18

**Authors:** Daniela Terracciano, Carlo Buonerba, Luca Scafuri, Piergiuseppe De Berardinis, George A. Calin, Alessandra Ferrajoli, Muller Fabbri, Amelia Cimmino

**Affiliations:** ^1^Department of Translational Medical Sciences, University Federico II, Naples, Italy; ^2^CRTR Rare Tumors Reference Center, AOU Federico II, Naples, Italy; ^3^Environment & Health Operational Unit, Zoo-Prophylactic Institute of Southern Italy, Portici, Italy; ^4^Institute of Biochemistry and Cell Biology, National Research Council, Naples, Italy; ^5^Department of Experimental Therapeutics, The University of Texas MD Anderson Cancer Center, Houston, TX, United States; ^6^Division of Cancer Prevention and Population Sciences, The University of Texas MD Anderson Cancer Center, Houston, TX, United States; ^7^University of Hawaii Cancer Center, Cancer Biology Program, Honolulu, HI, United States; ^8^Institute of Genetics and Biophysics, National Research Council, Naples, Italy

**Keywords:** coronavirus disease 2019 (COVID-19), cancer, severe acute respiratory syndrome coronavirus 2 (SARS-CoV-2), pandemic, chemotherapy

## Abstract

On March 11, 2020, the WHO has declared the coronavirus disease 2019 (COVID-19) a global pandemic. As the last few months have profoundly changed the delivery of health care in the world, we should recognize the effort of numerous comprehensive cancer centers to share experiences and knowledge to develop best practices to care for oncological patients during the COVID-19 pandemic. Patients as well as physicians must be aware of all these constraints and profound social, personal, and medical challenges posed by the tackling of this deadly disease in everyday life in order to adjust to such a completely novel scenario. This review will discuss facing the challenges and the current approaches that cancer centers in Italy and United States are adopting in order to cope with clinical and research activities.

## Introduction

Currently, the world is facing an ongoing pandemic of coronavirus disease 2019 (COVID-19) caused by severe acute respiratory syndrome coronavirus 2 (SARS-CoV-2). As of May 1, 2020, in Italy, the rapid spread of the virus had caused 204,576 confirmed cases (including 21,338 health care workers) and 26,049 deaths; the median age was 62 years (1). Very limited data are available for patients with cancer during the COVID-19 pandemic. Data on comorbidities from the Istituto Superiore di Sanità on April 23, 2020, described the characteristics of 2,041 patients with COVID-19 dying in the hospital for whom it was possible to analyze the clinical charts. Overall, only 3.6% of the sample presented with no comorbidities, 14.4% with a single comorbidity, 21.1% with two, and 60.9% with three or more comorbidities. In this series of fatal COVID-19 cases, 328 patients (16.1%) had been diagnosed with active cancer (1). The European Society for Medical Oncology recommends that clinical oncologists remain ready to adjust their routines. This suggests bolstering telemedicine services, reducing clinic visits, and switching to subcutaneous or oral therapies, rather than intravenous ones, when possible (2). There is also advice on supporting patients and infection control. Here, we report how several Italian and American cancer centers have reorganized their health care systems and updated their recommendations for testing patients with cancer, also identifying and discussing future research strategies.

## Coronavirus Disease 19 Triage Tests for Patients With Cancer: The Molecular Biologist Perspective

**FIGURE 1:**
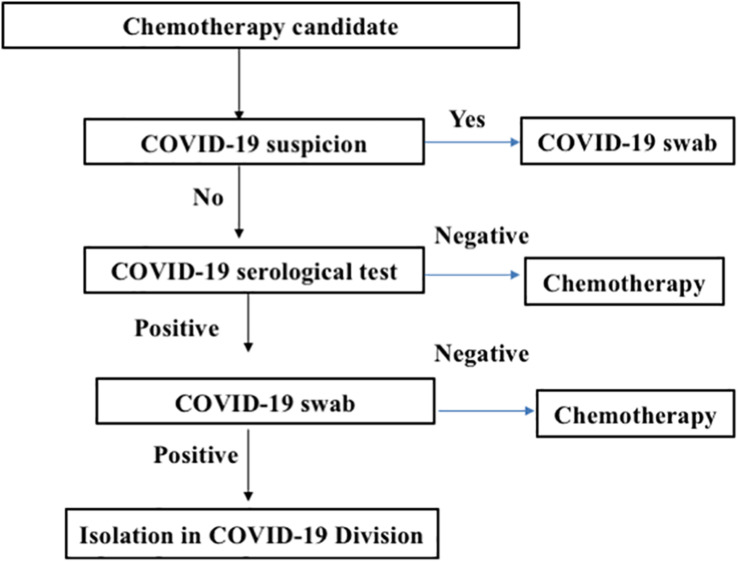
Triage screening for cancer patients before admission to the medical oncology ward. The flowchart describes the strategy of chemotherapy patient management to decrease the risk for treating coronavirus disease 2019 (COVID-19)-positive patients.

At University Federico II in Naples, the triage of an asymptomatic patient with cancer initially included a lateral flow chromatographic immunoassay (LFIA) for the qualitative detection of immunoglobulin (Ig)G and IgM antibodies against SARS-CoV-2 in fingerstick whole-blood specimens. LFIA positivity for IgM or IgG in symptomatic patients was further assessed *via* nasopharyngeal swabs and tested by real-time polymerase chain reaction (RT-PCR) for SARS-CoV-2 (Figure 1). The rapid LFIA test for IgM and IgG can complement the RT-PCR test and can be helpful, especially during the early phase of infection (3). Rapid antigen tests can theoretically provide the advantage of quicker time to results and low-cost detection but are likely to suffer from poor sensitivity (4–6). Recent reports from many European countries suggest that most of the rapid tests for COVID-19 did not show good sensitivity and did not detect over 70% of COVID-19 cases (7, 8). Collectively, data from the literature indicate that the COVID-19 IgM/IgG LFIA rapid test is not recommended for the triage of patients with suspected COVID-19. Nevertheless, Long et al. (9) recently demonstrated that IgG antibodies were detected in 100% of COVID-19 patients and that both IgM and IgG reached a plateau simultaneously or closely within 6 days after seroconversion. On this basis, serological tests may facilitate the identification of asymptomatic individuals infected with SARS-CoV-2. Therefore, a good serological test could be considered a very precious tool in COVID-19 triage in patients with cancer requiring active treatment. Chemiluminescence immunoassay (CLIA) has an exciting potential, running on an automated chemiluminescence analyzer with a throughput of about 50 tests/h for the detection of IgG and IgM in about 30 min (10, 11). Compared with rapid LFIA tests, the advantages of automated CLIA analyzers-based COVID-19 assays include not only the very high throughput of samples that can be analyzed but also the ability to perform tests for other biomarkers, such as C-reactive protein (CRP), which also needs to be monitored in COVID-19 suspects (12). Since CLIAs for IgM and IgG in venous blood were available at Federico II University, the approach changed as follows: (a) asymptomatic individuals underwent serological testing for IgG and/or IgM anti-SARS-CoV-2; and in case of positivity, RT-PCR for SARS-CoV-2 would be performed in respiratory tract specimens collected by nasopharyngeal swabs and (b) symptomatic individuals underwent nasopharyngeal swab collection and rapid RT-PCR for SARS-CoV-2 detection (13, 14) available at our university hospital.

## Ethical Dilemmas and Clinical Challenges: The Oncologist’s Perspective

Cancer patients are more likely to develop COVID-19, and cancer is associated with a more severe course of the disease (15); therefore, novel strategies to contain SARS-CoV-2 spreading among patients with cancer represent a compelling need in the current pandemic scenario.

One approach is to assess the feasibility, on a case-by-case basis, to postpone scheduled office visits of patients who are neither on active therapy nor on oral antineoplastic medication. The use of telephone, e-mail, or commercially available instant messaging applications may allow the treating oncologist to assess the reliability of referred symptoms and evaluate the results of prescribed tests on the grounds of the patient’s cancer history. Although digital technology has evolved to enable the transfer of hundreds of megabytes of data, as it is the case for computed tomography or magnetic resonance images using freely available online services, it would be more appropriate if dedicated digital platforms could be provided by health care providers along with standardized procedures in order to facilitate the process, protect privacy, and preserve data integrity. The initial evaluation of patients before they are admitted to the medical oncology ward is mandatory to identify those with a strong suspicion of being infected with SARS-CoV-2, who should first undergo nasal swab collection and can only be admitted after COVID-19 has been ruled out. The benefits associated with the use of rapid screening tests are hampered by their low accuracy, which carries potential harm associated with the performance of unnecessary tests and a potential delay in the delivery of antineoplastic therapy in false-positive patients, who have to wait for RT-PCR testing before they can be admitted.

Since most patients infected with SARS-CoV-2 are asymptomatic, the diagnosis of asymptomatic disease poses unresolved clinical challenges. Although most patients with severe COVID-19 are not eligible to receive antineoplastic therapy, the optimal management of cancer patients with asymptomatic or mild COVID-19 is debatable. Immunotherapy with anti-programmed cell death protein 1 (PD-1)/programmed cell death ligand 1 (PDL-1) agents may facilitate the progression to severe COVID-19 in asymptomatic SARS-CoV-2 patients according to the so-called “cytokine storm” theory (16). However, clinical data seem to show that COVID-19 patients are not at a greater risk of serious adverse events or a more aggressive course of the disease upon treatment with anti-PD-1/PDL-1 monoclonal antibodies (17). The clinical course of asymptomatic disease in patients with cancer is currently unknown; therefore, the risk of delaying antineoplastic therapy until the patient becomes SARS-CoV-2 negative cannot be accurately estimated, and a decision must be made on a case-by-case basis with the patient’s informed consent.

Compared with outpatients, inpatients must be informed of the additional risk associated with intra-hospital exposure to the virus and accept all procedures required by hospital policies (e.g., nasal swab). During the pandemic, patients with symptoms that may be associated with COVID-19 should undergo testing even if a more likely cause is identified (e.g., fever in neutropenic patients should prompt nasal swab collection, as well as cough in patients with interstitial pneumonia possibly associated with antineoplastic medication). At admission, patients should consent that any refusal to undergo SARS-CoV-2 testing, if deemed necessary by the hospital staff, would prompt immediate discharge or transfer to another ward.

Finally, one important aspect in clinical practice is the implications of physical distancing in the patient–physician relationship. Trust, which is the basis of a successful relationship, can easily be gained in some patients, while it is hard to achieve in others, especially with the current restrictions imposed due to the COVID-19 pandemic. Furthermore, the use of surgical masks during office visits may make it more difficult to interpret patients’ emotions, perplexities, and questions.

## Response to the Coronavirus Disease 2019 Pandemic at the University of Texas MD Anderson Cancer Center, Houston

Starting in February 2020, several nations implemented various measures to reduce the spread of COVID-19. The medical community found itself in a “state of emergency” due to the rapid spreading of a pandemic infection caused by a novel coronavirus, SARS-CoV-2. With limited prior experience in the management of a pandemic, very limited testing options, and rudimental knowledge of this new viral disease, hospitals around the world designed isolation, testing, and treatment strategies to best protect their patient populations.

At our tertiary cancer center, current strategies focus on preventing exposure, expanding testing, and implementing hospital-wide algorithms for the supportive care and treatment of affected patients. These changes have a significant impact on daily clinical activities and the choice of treatments for patients with cancer.

### Clinical Activities

In order to prevent the spread of the infection, hospital admissions and visits to the clinic are limited to patients in need of emergent and urgent treatments. Clinical areas are made accessible only to essential clinical personnel; in-person appointments are confirmed only for patients who require initial evaluation for an urgent or emergent cancer diagnosis and for patients who need to continue life-saving ongoing cancer therapies. The remaining patients are assisted by a variety of strategies that include telephone appointments, virtual care visits, and close partnership with oncologists and primary care physicians in the community. Our center is a research institution, and many patients are receiving treatments within clinical trials. In compliance with the regulatory authorities, each clinical study implemented a plan to monitor enrolled participants to ensure safety and to obtain the clinical information to be able to continue to evaluate the investigated intervention activity.

We invested a significant effort in educating our patients regarding the risk of contracting the SARS-CoV-2 infection. All patients received education materials digitally, and additional information was provided in person at the time of clinic visits. Patients with cancer are aware of being susceptible to infections and are accustomed to taking precautions; therefore, we have seen excellent adherence to our recommendations and a low rate of SARS-CoV-2 positivity in our patient population.

Prior to entering the clinic, all patients (as well as staff) go through an interview process that assesses the presence of symptoms and history of exposure, have a body temperature check, perform hand sanitation, and are provided with a mask. Patients suspected of having the infection are screened, triaged, evaluated, and treated by dedicated medical teams equipped with appropriate protective gear and kept physically separated from the rest of the patients until their testing results become available. Patients with positive COVID-19 tests are transferred to a specialized multidisciplinary team.

The most widely used testing tool at our center has so far been RT-PCR testing for the presence of SARS-CoV-2 in samples obtained through a posterior pharynx nasal swab. Simultaneous testing for SARS-CoV-2 and other common respiratory viruses is routinely performed. Our institution has recently expanded RT-PCR testing to all patients immediately prior to undergoing surgery or other invasive procedures or receiving intensive chemotherapy regimens and cellular therapies. Two additional testing modalities are key to a timely and complete patient assessment: a rapid antigen assay and IgG and IgM antibody titers evaluation to follow the development of SARS-CoV-2 antibody response. We anticipate that a rapid antigen assay should be available in the near future and will be used as a screening tool. Antibody testing has recently become available in our institution for selected patients, and it is likely to be used to screen health care workers once supply is in sufficient amount and the test has been validated for its accuracy.

### Treatment Planning

The ongoing COVID-19 pandemic and the individual patient risk of developing severe disease are additional elements that need to be considered during the development of an oncology treatment plan. Several reports have described a higher mortality in patients with cancer who contracted COVID-19; however, the majority of the reports have small sample sizes, and the population described is heterogeneous in terms of cancer stage, treatment, and the presence of other contributing factors such as comorbidities. The recommendations implemented at our center are based on the consensus opinion of disease experts and focus on reducing the number of days with myelosuppression by preferring less myelosuppressive regimens (when an alternative treatment is available), administering growth factors, temporarily discontinuing treatments for patients in remission with low risk for relapse, and optimizing antimicrobial prophylaxis and supportive care. An open discussion on the risks and benefits, individual patient goals, and where each patient is in their cancer disease journey, combined with disease-specific metrics, is very important not only to help patients and their families in making an educated decision but also to the institution in its effort to provide high-quality, appropriate, and compassionate cancer treatment at a time of need to prioritize available resources.

There is a possibility that certain cancer treatments may have beneficial effects in patients who have contracted COVID-19 and have respiratory complications. In a recently published letter, Treon et al. (18) reported a beneficial effect of treatment with the Bruton’s tyrosine kinase (BTK) inhibitor ibrutinib on lung inflammation and hypoxia in five patients with Waldenström macroglobulinemia. This lead has been followed by other groups that have reported the potential protective effect of acalabrutinib in patients with lymphoproliferative disorders affected by severe forms of COVID-19 (19) and by the initiation of clinical trials with BTK inhibitors in patients with severe COVID-19. The mechanisms underlying the activity of BTK inhibitors in improving hypoxia in patients with severe COVID-19 seem to be mediated by an inhibition of exaggerated monocyte autophosphorylation and reduction in interleukin (IL)-6 production.

Cancer centers like ours have also developed tools to assist in the care of patients affected by COVID-19. The first step is deciding which patients need to be treated in the inpatient setting and which can safely remain outpatients. Cancer diagnosis, current treatment, age, gender, and comorbidities are key elements in this initial decision. Symptomatic patients who require in-hospital care are risk-stratified based on the severity of illness and managed according to an internal COVID-19 clinical management algorithm compiled by a multidisciplinary task force. This algorithm has been revised and updated multiple times to incorporate the most recent information available on COVID-19 features and treatment in patients with cancer and relevant publications and consensus statements from the Centers for Disease Control and Prevention (CDC), various medical societies, and task forces. The goal of the algorithm is to provide both guidance and uniformity in diagnostic workup, supportive care, and treatment of patients affected by COVID-19, given the unprecedented challenges that this disease poses and the absence of consensus guidelines.

## Changes in Cancer Care in the Coronavirus Disease 2019 Era: Illustrative Clinical Scenarios and Potential Differences Between Italian and United States-Based Centers

In some clinical settings, both oral and intravenous agents are indicated. For example, in prostate cancer patients with metastatic castration-sensitive disease, both novel hormonal agents including abiraterone, apalutamide, and enzalutamide and chemotherapy-based docetaxel have been shown to provide a survival advantage (20). While in the universal Italian health care system, an oral hormonal agent is more likely to be preferred over docetaxel in view of a more favorable safety profile as well as reduced risk of hospitalization and need for in-person visits, some patients in the US may not afford such an expensive and prolonged treatment and prefer a short course of docetaxel. Similarly, in kidney cancer, oral agents including cabozantinib, sunitinib, and pazopanib may be preferred over nivolumab/ipilimumab (21). In clinical scenarios in which adjuvant treatment benefit is uncertain, such as muscle-invasive bladder cancer after radical cystectomy, the COVID-19 emergency may discourage the use of cisplatin-based chemotherapy (22).

While there are multiple clinical situations in which an oral treatment may be preferred over intravenous therapy, accessibility, reimbursement policies, and overall costs may cause differences in Italian vs. United States-based clinical practices.

## Cancer Immunologists Combating Biological Mechanisms in Coronavirus Disease 2019

Since November 2019, we have been exposed to a global outbreak recalling the 1918 Spanish flu and facing similar challenges and solutions, such as wearing safety devices and observing physical distancing. However, we have an important advantage, as we have a much deeper knowledge of the immune system, thanks to years of studies and research achievements in the context of infections, autoimmune diseases, and cancer. Particularly in the last two decades, we have experienced the flourishing and rapid evolution of immunotherapeutic strategies of high impact in oncology. Knowing that the immune response plays a central role in the pathogenetic mechanisms of COVID-19, we can thus exploit all the experience accumulated in recent years to better understand the constellation of symptoms of a disease that attacks almost any organ with devastating consequences in roughly 5% of patients. As an example, we recently learned that a rare disorder named secondary hemophagocytic lymphohistiocytosis, previously associated with viral infections and T-cell immunotherapy-based cancer treatments, was identified in some COVID-19 patients (23).

The varied symptoms observed in COVID-19 patients often appear as a consequence of an overzealous immune response. The high cytokine serum levels, such as IL-6 and IL-1, reflect the “cytokine storm,” a familiar event to oncologists, seen in patients with cancer who receive chimeric antigen receptor (CAR) T-cell immunotherapy and, thanks to this knowledge, efficacious treatments with anti-IL-6 monoclonal antibodies have been developed. However, since cytokines are a necessary feature of the antiviral immune responses, what are the consequences of inhibiting them with immunosuppressive treatments?

Concerning cellular immunity, it is fundamental to know the role played by different cell populations in COVID-19. In other words, which cells are helpful and which are hurtful. Some evidence suggests that T cells, which usually represent a key component of the immune response against viruses, do not play a role in lung damage during COVID-19 infection, while stimulation of other cells in the lung microenvironment, such as macrophages, may make a difference.

All these important questions, with relevant implications for treatment options, need answers that we are eager to find for the benefit of society. In this context, we are aware that the public often suffers from a high dose of misinformation from social media. In addition, an overwhelming amount of discordant or badly reported scientific results often cause great concern and alarm in the public opinion. For this reason, correct information, also aimed at a lay audience, is important. In this context, we recently organized a website named InformaCOVID^1^, open to contributions from experts dealing with COVID-19-related topics in a popular style of writing. The site has had many visitors so far, and we hope it will remain an important instrument for accurate scientific information.

## Coronavirus Disease 2019 Impact on Cancer Research in the United States

The rapid emergence of the COVID-19 pandemic has forced cancer research institutions worldwide to make decisions that prioritize the safety of their scientists and administrators and the need to continue ongoing experiments and meet timelines and deadlines. In the United States, we have faced a whole spectrum of solutions ranging from a complete shutdown of any cancer research activity and lockdown of research labs to more compromising situations in which scientists are performing experiments, minimizing the amount of time they spend next to each other. This last goal is achieved by creating work shifts that limit the number of scientists present at the same time in the lab to a maximum of two (wearing face masks and gloves all the time and maintaining a 6-feet distance to each other), reducing the number of people working with cell cultures under the hood or in smaller rooms to one, not allowing in-person meetings with more than 10 people in general, and implementing a 6-feet social distancing practice even when 10 or fewer people are allowed to meet in person. Additionally, all work-related activities that do not require performing an experiment in a wet lab are encouraged to be performed at home, by implementing a “work-from-home” policy. *In vivo* experiments have been quite impacted because of a reduction of personnel working on animal care during this period; given that animal experiments are usually quite expensive, complex, and long-lasting, principal investigators (whenever possible) are forced to postpone them until regular activities resume.

The National Institutes of Health (NIH) has been promptly responsive and has implemented a series of guidelines and initiatives to ensure that research continues. The NIH has created new funding opportunities through competing and administrative supplements to investigators with currently ongoing NIH support and in the creation of new awards (ongoing funding opportunities can be found at https://grants.nih.gov/policy/natural-disasters/corona-virus.htm). The National Cancer Institute (NCI) is participating in five out of the 45 NIH funding opportunities specific to COVID-19. Specifically, the NCI participates in **NOT-CA-20-054** (“Notice of Information: Contributing to the Global COVID-19 Crisis Response by Allowing Some NCI-Supported Projects to Be Redirected to COVID-19-Related Research During the Crisis”), **NOT-RM-20-015** [“Notice of Special Interest (NOSI): Availability of Emergency Competitive Revisions for Research on Severe Acute Respiratory Syndrome Coronavirus 2 (SARS-CoV-2) and COVID-19”], **NOT-OD-20-097** (“NOSI regarding the Availability of Administrative Supplements and Urgent Competitive Revisions for Research on the 2019 Novel Coronavirus and the Behavioral and Social Sciences”), **NOT-CA-20-042** (“NOSI: National Cancer Institute Announcement Regarding Availability of Urgent Competitive Revision and Administrative Supplements on COVID-19”), and **NOT-CA-20-043** (“NOSI: National Cancer Institute Announcement Regarding Availability of Competitive Revision SBIR/STTR Supplements on COVID-19”). Moreover, the NIH has shown some administrative flexibility in deadline submissions and has published guidelines on human research, animal research, and accommodations for loss of research time. These measures are certainly of great help to lessen the bureaucratic burden during this period and foster new committed support to promote the rapid development of new ideas to contrast this unprecedented pandemic in general and its specific impact on cancer patients.

Another challenge that the research community is facing is the impossibility of holding in-person scientific meetings. The opportunity to interact with peers and present original data to the scientific community can only in part be replaced by “virtual” meetings. However, the rapid adaptation of the main scientific societies to web-based platforms has been extremely helpful in minimizing the impact of travel restrictions on the sharing of research findings, which remains essential for the scientific community.

## Concluding Remarks

The lessons we are learning during the response to the COVID-19 pandemic will likely accelerate the integration of digital technologies in the care of patients with cancer. Cancer centers worldwide have expanded their telemedicine capabilities very rapidly, and we anticipate an increasing use of telemedicine for the long-term follow-up of patients not on active therapies, for patients in remote locations, and for multidisciplinary case planning and tumor boards.

An organized triage and screening tests are essential for the safe management of patients with cancer during the ongoing COVID-19 pandemic. Patients with cancer have shown an excellent adherence to the recommended preventive measures to reduce the risk of infections, and the SARS-CoV-2 positivity rate seen at a major cancer center is lower than the rate reported in the general population. It is important to provide clear and validated information to patients with cancer and their families to optimize preventive strategies during the COVID-19 pandemic.

Concerning research work, lockdown decisions aimed at prioritizing safety have seen a partial block of entry in research institutions with drastic reductions in wet lab experiments and preclinical animal model-based studies. However, some institutions, such as the NIH, have been promptly responsive and implemented guidelines to allow for the continuation of research activities. In addition, new funding opportunities have been created. Finally, the flourishing of virtual meetings has provided opportunities for many scientists to interact with peers, discuss, and better organize their research, opportunities that should be kept after the pandemic.

## Author Contributions

AC and DT contributed to the conception of the work and final version approval. GC, MF, CB, LS, PD, and AF contributed to extensive literature search, manuscript drafting, and critical revision of the work. All authors contributed to the article and approved the submitted version.

## Conflict of Interest

The authors declare that the research was conducted in the absence of any commercial or financial relationships that could be construed as a potential conflict of interest.

## Footnotes

1 www.informaCOVID.wordpress.com

## References

[1] EpiCentro *L’Epidemiologia per la Sanità Pubblica: Istituto Superiore di Sanità.* (2020) Available online at: https://www.epicentro.iss.it/coronavirus/bollettino/Report-SARSCOV-2_30_marzo.pdf (accessed May 1, 2020)

[2] European Society for Medical Oncology (ESMO) *The ESMO-Magnitude of Clinical Benefit Scale.* (1995) Available online at: https://www.esmo.org/guidelines/esmo-mcbs/esmo-magnitude-of-clinical-benefit-scale (accessed April 2, 2020).

[3] Padoan A Cosma C Sciacovelli L Faggian D Plebani M. Analytical performances of a chemiluminescence immunoassay for SARS-CoV-2 IgM/IgG and antibody kinetics. *Clin Chem Lab Med.* (2020) 58:1081–88. 10.1515/cclm-2020-0443 32301749

[4] Nationalreview *China Supplied Faulty Coronavirus Test Kits to Spain, Czech Republic.* (2020) Available online at: https://www.nationalreview.com/news/china-supplied-faulty-coronavirus-test-kits-to-spain-czech-republic/ (accessed April 2, 2020).

[5] Businessinsider *Spain, Europe’s Worst-Hit Country after Italy, Says Coronavirus Tests It Bought from China are Failing to Detect Positive Cases.* Available online at: https://www.businessinsider.com/coronavirus-spain-says-rapid-tests-sent-from-china-missing-cases-2020-3?IR=T.

[6] PragueMorning *80% of Rapid COVID-19 Tests the Czech Republic Bought From China are Wrong.* (2020) Available online at: https://www.praguemorning.cz/80-of-rapid-COVID-19-tests-the-czech-republic-bought-from-china-are-wrong/ (accessed April 2, 2020).

[7] FoxNews *Netherlands Becomes Latest Country to Reject China-Made Coronavirus Test Kits, Gear.* (2020) Available online at: https://www.foxnews.com/world/netherlands-becomes-latest-country-to-reject-china-made-coronavirus-test-kits-gear (accessed April 2, 2020).

[8] Cassaniti I Novazzi F Giardina F Salinaro F Sachs M Perlini S Members of the San Matteo Pavia COVID-19 task force. Performance of VivaDiag COVID-19 IgM/IgG Rapid Test is inadequate for diagnosis of COVID-19 in acute patients referring to emergency room department. *J Med Virol.* (2020) 10.1002/jmv.25800 [Epub ahead of print]. 32227490 PMC7228409

[9] Long Q Liu BZ Deng HJ Wu GC Deng K Chen YK Antibody responses to SARS-CoV-2 in patients with COVID-19. *Nat Med.* (2020) 26:845–48. 10.1038/s41591-020-0897-1 32350462

[10] Snibe *The World’s First 2019-nCoV CLIA Kits Received CE Mark.* (2017) Available online at: http://www.snibe.com/zh_en/en_newsView.aspx?id=576 (accessed April 2, 2020) p. 291–99. 10.4324/9780429453885-9

[11] Infantino M Grossi V Lari B Bambi R Perri A Manneschi M Diagnostic accuracy of an automated chemiluminescent immunoassay for anti-SARS-CoV-2 IgM and IgG antibodies: an Italian experience. *J Med Virol.* (2020) 10.1002/jmv.25932 [Epub ahead of print]. 32330291 PMC7264663

[12] Lippi G Salvagno GL Pegoraro M Militello V Caloi C Peretti A Assessment of immune response to SARS-CoV-2 with fully automated MAGLUMI 2019-nCoV IgG and IgM chemiluminescence immunoassays. *Clin. Chem. Lab. Med.* (2020). 58:1156–59. 10.1515/cclm-2020-0473 32301750

[13] Loeffelholz MJ Tang YW. Laboratory diagnosis of emerging human coronavirus infections–the state of the art. *Emerg Microbes Infect.* (2020) 9:747–59. 10.1080/22221751.2020.1745095 32196430 PMC7172701

[14] Rhoads DD Cherian SS Roman K Stempak LM Schmotzer CL Sadri N. Comparison of Abbott ID Now, diasorin simplexa, and CDC FDA EUA methods for the detection of SARS-CoV-2 from nasopharyngeal and nasal swabs from individuals diagnosed with COVID-19. *J Clin Microbiol.* (2020).58:e00760-20. 10.1128/JCM.00760-20 32303564 PMC7383529

[15] Wang H Zhang L. Risk of COVID-19 for patients with cancer. *Lancet Oncol.* (2020) 21:e181 10.1016/s1470-2045(20)30149-2 PMC7129735 32142621

[16] Chen G Wu D Guo W Cao Y Huang D Wang H Clinical and immunologic features in severe and moderate Coronavirus Disease 2019. *J Clin Invest.* (2020) 130 2620–29. 32217835 10.1172/JCI137244 PMC7190990

[17] Luo J Rizvi H Egger JV Preeshagul IR Wolchok JD Hellmann MD. Impact of PD-1 blockade on severity of COVID-19 in patients with lung cancers. *Cancer Discov.* (2020) 10:1121–1128. 10.1158/2159-8290.CD-20-0596 32398243 PMC7416461

[18] Treon S. P. Castillo JJ Skarbnik AP Soumerai JD Ghobrial IM Guerrera ML The BTK inhibitor ibrutinib may protect against pulmonary injury in COVID-19 – infected patients. *Blood.* 135 1912–1915 (2020). 10.1182/blood.2020006288 32302379 PMC7243149

[19] Roschewski M Lionakis MS Sharman JP Roswarski J Goy A Monticelli MA Inhibition of bruton tyrosine kinase in patients with severe COVID-19. *Sci Immunol*. (2020) 5:eabd0110. 10.1126/sciimmunol.abd0110 32503877 PMC7274761

[20] Buonerba C Ferro M Dolce P Crocetto F Sonpavde G. Predictors of efficacy of androgen-receptor-axis-targeted therapies in patients with metastatic castration-sensitive prostate cancer?: a systematic review and meta-analysis. *Crit Rev Oncol.* (2020) 151:102992. 10.1016/j.critrevonc.2020.102992 32474391

[21] Buonerba C Dolce P Iaccarino S Scafuri L Verde A Costabile F Outcomes associated with first-line anti-PD-1/PD-L1 agents vs. sunitinib in patients with sarcomatoid renal cell carcinoma: a systematic review and meta-analysis. *Cancers.* (2020) 10:408. 10.3390/cancers12020408 32050629 PMC7072485

[22] Zhegalik AG Polyakov SL Rolevich AI Volkov AN Minich AA Vasilevich VJ Long-term results of a single-center prospective randomized trial assessing efficacy of a shortened course of adjuvant chemotherapy after radical cystectomy in patients with locally advanced bladder cancer. *Cent Eur J Urol.* (2020) 73:26–32. 10.5173/ceju.2020.0032 32395319 PMC7203780

[23] Mehta P McAuley DF Brown M Sanchez E Tattersall RS Manson JJ COVID-19: consider cytokine storm syndromes and immunosuppression. *Lancet.* (2020) 395:1033–34. 32192578 10.1016/S0140-6736(20)30628-0 PMC7270045

